# Psychometric Properties of the Hindi Version of the Disabilities of Arm, Shoulder, and Hand: A Pilot Study

**DOI:** 10.1155/2015/482378

**Published:** 2015-12-31

**Authors:** Saurabh P. Mehta, Ramesh Tiruttani, Manraj N. Kaur, Joy MacDermid, Rania Karim

**Affiliations:** ^1^School of Physical Therapy, Marshall University, Huntington, WV, USA; ^2^Department of Orthopedics Surgery, Marshall University, Huntington, WV, USA; ^3^Shree Swaminarayan College of Physiotherapy, Surat, India; ^4^School of Rehabilitation Science, McMaster University, Hamilton, ON, Canada; ^5^Department of Surgery, Surgical Outcomes Research Center, Hamilton, ON, Canada

## Abstract

*Objectives*. To culturally adapt and translate the Disabilities of Arm, Shoulder, and Hand questionnaire into Hindi (DASH-H) and assess its reliability, validity, and responsiveness in adult patients with shoulder tendonitis.* Study Design*. Descriptive methodological research, using longitudinal design.* Setting*. Outpatient clinic.* Participants*. 30 adult patients aged 53.3 ± 6.9 y with shoulder tendonitis.* Data Analyses*. DASH-H, visual analogue scales for pain (VAS-P) and disability (VAS-D), and shoulder active range of motion (AROM) were assessed at baseline, 2-3 days later, and 4-5 weeks after baseline. Intraclass correlation coefficients (ICC) assessed test-retest reliability of these scales and responsiveness was examined by calculating effect sizes (ES) and standardized response means (SRM). Cronbach's alpha (CA) was used to examine internal consistency of DASH-H. Convergent construct validity of DASH-H with VAS scales and shoulder AROM was determined using Pearson's Correlation Coefficients (*r*).* Results*. DASH-H demonstrated good test-retest reliability and internal consistency (ICC and CA both > 0.75) and excellent responsiveness (ES = 2.2, SRM = 6.1). DASH-H showed high concordance (*r* = −0.71, *p* < 0.01) with AROM-flexion and moderate concordance (*r* > −0.4, *p* < 0.05) with VAS scales and AROM-external rotation.* Conclusion*. Analyses indicate that DASH-H demonstrates good test-retest reliability, validity, and responsiveness in patients with shoulder tendonitis.

## 1. Introduction

Patient-reported outcome measures are routinely used in determining the impact of a condition on the health status of an individual [1]. Patient-reported outcome (PRO) measures provide the assessments of aspects of impairments experienced by the patients which cannot be examined directly by the clinicians. The clinical utility of patient-reported outcome measures is examined by the existing evidence regarding its measurements properties such as reliability, validity, and sensitivity to change [2].

Since its inception almost 2 decades ago [3], the Disabilities of Arm, Shoulder, and Hand (DASH) questionnaire has become an extremely useful PRO for assessing upper extremity (UE) disability in individuals with various UE musculoskeletal conditions. Numerous studies have supported the reliability, validity, and sensitivity to change of the DASH across a variety of UE musculoskeletal conditions [4–7]. The DASH consists of 30 items which provide an overall assessment of upper extremity disability. In 2005, a shorter 11-item version of the DASH, known as the QuickDASH, was conceived to reduce the administrative burden yet retaining the main aspects of upper extremity functions in assessing disability [8]. A recent review suggested that while there might be inadequate evidence regarding the sensitivity to change, the QuickDASH has overall demonstrated excellent reliability and validity across several upper extremity conditions [9]. In general, the QuickDASH is recommended over the full length DASH for assessing UE disability in clinical practice. However, the full length DASH is suggested for use in a research study [9].

Patient-reported outcome measures require translations and cultural adaptations when used in a language or a culture other than the language or culture they were originally developed in. The translation and cultural adaptation procedures have the purpose of semantic and experiential equivalence for successful integration in the target population [10]. The DASH has been translated and culturally adapted for use in over 45 languages/cultures [11]. The developers of the original DASH provided standardized stepwise guidelines to which researchers should adhere when translating the questionnaire into their target language [12]. The researchers are required to adhere to these guidelines. This method increases the internal validity of the translation and cultural adaption process.

Hindi is the official language of the government of India and one of the most common languages spoken worldwide. The majority of people who speak Hindi live in India; however, a number of people who have migrated to North America, Europe, or parts of Australia consider Hindi as their native language. In the recent past, several outcome measures have been translated for use in Hindi speaking patient population [13–17]. However, no Hindi version currently exists for the DASH. This may lead to rehabilitation practitioners not being able to adequately capture functional disability of Hindi speaking individuals with upper extremity pathology. The literature regarding the prevalence of UE musculoskeletal pathologies in India is limited, with the prevalence only being determined for work-related UE conditions [18, 19]; it can be assumed that the number of individuals with UE musculoskeletal conditions will be substantial in a country of 1.2 billion people. A Hindi version of the DASH can not only provide a useful measure for practitioners across the world treating Hindi speaking individuals with UE musculoskeletal problems but also facilitate outcome measurement across research trials involving such participants.

Therefore, the present study had two objectives. In the first step, cultural adaptation and translation of the DASH into Hindi were performed. In the second step, preliminary assessment of the reliability, validity, and responsiveness was conducted for the prefinal version of the DASH-Hindi (DASH-H) in patients with shoulder tendonitis.

## 2. Methods

### 2.1. Cross-Cultural Adaptation

Standardized guidelines for performing linguistic translation and cultural adaptation of an outcome measure have been published by the developers of the DASH [10]. The 5-step process recommended in these guidelines was followed. Forward translation of the DASH into Hindi was conducted by two retired university professors in India whose native language was Hindi. This resulted in two versions of the DASH-H (DASH-H1 and DASH-H2). One of the two translators was the head of English department in a government university at the time of retirement and the other was the professor in the department of physics at the same university. Therefore, none of them had any background in health sciences which ensured that the forward translation was performed using a language that would be understandable to a lay person.

Both translators, a clinician and one of the authors of this study (SM), met to work on the two forward translations (DASH-H1 and DASH-H2) and prepared the mutually agreeable synthesized version of the DASH-H (DASH-H12). Subsequently, this synthesized Hindi version was translated back to English (DASH-H BT1 and BT2) by two separate translators to screen whether the content on these translated versions was representative of the original DASH. The expert committee consisting of researcher, clinician, linguistic expert, and translators met with the objectives of reviewing the synthesized version item-by-item, further refining the synthesized version and creating the prefinal version of the DASH-H.

### 2.2. Pilot Testing the Psychometric Properties of the DASH-H

Once the expert committee approved the prefinal version of the DASH-H, preliminary analysis of its psychometric properties was conducted.

#### 2.2.1. Study Participants

Consecutive patients diagnosed with shoulder tendonitis presenting to the outpatient rehabilitation clinic affiliated with Shree Swaminarayan Physiotherapy College (SSPTC), Surat, India, between November 2010 and September 2011 were asked to participate in the study. Informed consent was sought from the study participants before data collection. The inclusion criteria were the following: being diagnosed with shoulder tendonitis, being able to read and comprehend Hindi, and being 18 years old or older. The shoulder tendonitis was diagnosed by the referring physician. Patients typically had pain around rotator cuff tendon which exacerbated with overhead shoulder movements and varying levels of restriction of shoulder joint range of motion (ROM). This patient group was chosen mainly to maximize recruitment, since recruiting a patient group with specific pathology would have slowed the recruitment process. Participants with concurrent injuries or preexisting musculoskeletal conditions in either of the upper extremities, those with neurological conditions, and those who were not able to attend all the three data collection sessions were excluded from the study. The research ethics board at McMaster University approved this study.

#### 2.2.2. Tests and Measures

*Disabilities of Arm, Shoulder, and Hand*. The DASH consists of 30 items inquiring about an individual's functional status in presence of upper extremity musculoskeletal condition. Each item is rated on the scale of 1–5 with 1 indicating no problem and 5 indicating extreme problems or inability to perform the activity. The total score on the DASH is derived by using a predefined formula and can range from 0 to 100 with 0 indicating no functional disability and 100 indicating worst disability. No more than 3 items can be left unanswered to accurately calculate the score on the DASH. The full length DASH-H was administered to the participants where they were asked to read and complete the questionnaire. If an item on the DASH-H was unclear, participants had the opportunity to ask for clarification of its meaning.

*Visual Analogue Scale (VAS).* A 0–10 cm VAS was administered to examine perceived pain (VAS-P) and disability (VAS-D). The anchors for both VAS were provided in Hindi with 0 indicating no pain or no disability and 10 indicating extreme pain or extreme difficulty. The reliability, validity, and responsiveness of VAS-P and VAS-D with Hindi anchors were shown to be excellent [13].

*Shoulder Joint Active ROM (AROM).* Shoulder AROM in flexion, abduction, and external rotation were measured for the affected side. A 12-inch 180° Stainless Steel Universal Goniometer (Indian Surgical Instruments Co., Jalandhar, India) was used for the assessment. The measurements for the shoulder flexion and abduction were performed with shoulder in neutral position and 0° abduction/adduction, respectively. The participants were in supine position. This is one of the most commonly used techniques for assessing the shoulder AROM in clinical practice and has been shown to be highly reliable [20].

#### 2.2.3. Data Collection Protocol

The second author of this paper (Ramesh Tiruttani) who was a faculty member and attending physiotherapist at the SSPTC collected all the study data. Upon agreeing to participate in the study, each participant completed the DASH-H and VAS scales. The assessor (Ramesh Tiruttani) obtained the measurements for the shoulder AROM. All these measures were administered again at 2-3 days and 4-5 weeks following the initial assessment. Patients continued to receive physical therapy treatment for their shoulder problem at the outpatient clinic affiliated with the SSPTC; however, the study investigators had no role in designing and administering any interventions.

### 2.3. Data Analysis

Descriptive statistics including mean and standard deviations (SD) for the continuous variables and frequency count (%) for the categorical variables were calculated. Floor and ceiling effects for the DASH-H were calculated by determining the percentage of participants with the scores between 0–10 and 91–100, respectively, at the baseline assessment.

Intraclass correlation coefficients (ICC) were calculated to examine the test-retest reliability of the DASH-H scores obtained at baseline and 2-3 days following that. ICC values of >0.75 were considered to be indicative of good reliability [21]. Cronbach's alpha (CA) was calculated as the measure of internal consistency of the DASH-H. The CA values of >0.75 were deemed to be indicative of good internal consistency. Pearson's Correlation Coefficients (*r*) were calculated for examining the convergent construct validity of the DASH-H with the VAS scales and shoulder AROM. Since the DASH-H measures shoulder disability across a range of functional tasks versus single item questions (VAS scales) or excursion in single shoulder movement (AROM), moderate concordance was expected between the DASH-H, VAS scales, and all three shoulder AROM assessed with *r* values ranging between 0.4 and 0.7.  The responsiveness of the DASH-H and VAS scales was examined by calculating the effect size (ES) and standardized response means (SRM). The ES was calculated by dividing the mean change between occasion 1 and occasion 3 by the standard deviation of the baseline scores of the measures. The SRM was calculated by dividing the mean change between occasion 1 and occasion 3 by the standard deviation of the change scores. The ES and SRM outcomes of >1 were considered to reflect excellent responsiveness [22, 23]. The standard error of measurement (SEM) was calculated using the formula SEM = SD ×  √1−ICC to indicate the random error associated with a single score. Lastly, the minimal detectable change (MDC) was calculated for the 90% (MDC_90_) and 95% (MDC_95_) confidence levels for both the DASH-H and VAS scales.

*p* values of ≤ 0.05 were considered as significant for all the analyses. SPSS (v.21, Chicago, IL) was used for conducting the data analysis.

## 3. Results

### 3.1. Cross-Cultural Adaptation

A few issues concerning the conceptual and experiential equivalence were resolved while preparing the synthesized and the prefinal versions of the DASH-H. Firstly, for example, the forward translators had selected separate Hindi words “
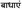
” and “
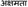
” for the word “difficulty.” The translators agreed to proceed with the word “
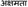
” since it was deemed to better represent “difficulty.” Secondly, the word “
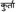
” which is ethnic apparel in India worn in the same manner as pullover sweater was added to item 15. The intent was to provide a better context to Hindi speaking individuals when responding to this item. One of the key issues was the fact that many words described in the English version of the DASH are now part of conversational Hindi. Either there are no equivalent Hindi words for them or the use of the translated Hindi words has almost become obsolete. Therefore, it was impractical to use Hindi translation for such words. Some of the examples are score, badminton, light bulb, and briefcase. While communicating in Hindi, these words are spoken as they are spoken in English. The translators agreed to keep these words as they are in the English version but use Hindi script for them. The prefinal version of the DASH-H was created after these deliberations in the expert committee meeting.

### 3.2. Pilot Testing of the Psychometric Properties of the DASH-H

#### 3.2.1. Descriptive Statistics

Table 1 shows the characteristics of 30 participants (16 females and 14 males) enrolled in this study. Seven participants were lost to follow-up and did not complete the third data collecting session. The main reason for loss to follow-up was participants experiencing pain relief and not requiring care beyond 2-3 weeks after baseline assessment. Twenty-six patients (87%) were right-hand-dominant and four patients (13%) were left-hand-dominant. No floor/ceiling effects were evident for the DASH-H. Participants reported significantly lower DASH scores (*p* ≤ 0.05) at occasion 2 and occasion 3 when compared to baseline measurements.

#### 3.2.2. Test-Retest Reliability and Internal Consistency

Table 2 shows the results of the test-retest reliability, assessed between occasion 1 and occasion 2, and the internal consistency. The test-retest reliability was assessed for all the measures, whereas the internal consistency was assessed only for the baseline DASH-H scores. The DASH-H demonstrated both good test-retest reliability and internal consistency (ICC and CA both >0.75). For internal consistency, Cronbach's alpha was 0.87 with values >0.75 being indicative of good internal consistency. Of the other measures, VAS-D and AROM for shoulder external rotation (ER) had good test-retest reliability (ICC ≥ 0.75).

#### 3.2.3. Convergent Construct Validity

Table 3 shows the results of convergent construct validity for the DASH-H with the other measures. The DASH-H showed high concordance (*r* = −0.71) with the AROM-flexion and moderate concordance with the VAS scales (*r* > 0.4) and AROM-external rotation (*r* = −0.47).

#### 3.2.4. Responsiveness

Table 4 shows the responsiveness statistics for the DASH-H and other measures. The DASH-H scores changed significantly from occasion 1 to occasion 3 suggesting change in the status (change of 31.9 ± 5.2; *p* < 0.001). The responsiveness of the DASH-H was shown to be excellent as observed by the ES and SRM values of 2.2 and 6.1, respectively. Similarly, the VAS-P also showed excellent responsiveness as seen by the ES and SRM values of 3.8 and 3, respectively. Lastly, the responsiveness of the VAS-D was also excellent as seen by the ES and SRM values of 2.64 and 2.73, respectively. The SEM for the DASH-H was 5.3 points, whereas MDC_90_ and MDC_95_ were 12.4 and 4.7 points, respectively. The SEM, MDC_90_, and MDC_95_ values for the other measures are also shown in Table 4.

## 4. Discussion

This study provides preliminary data examining the psychometric properties of the DASH-H in adult patients diagnosed with shoulder tendonitis. Results of the study support our initial hypotheses and demonstrate that the DASH-H has good test-retest reliability, construct validity, and responsiveness in this patient population. Current patient assessment approaches as well as research studies conducted on Hindi speaking individuals with UE musculoskeletal pathologies rely heavily on subjective information and objective information such as AROM and pain and not on standardized patient-reported outcome measures. Translation of the DASH into Hindi addresses this gap by proving an additional tool for researchers and clinicians to utilize. Moreover, this study will also facilitate future lines of research to further examine the psychometric properties of the DASH-H across different patient subgroups with upper extremity pathology. The study results were limited by the small sample recruited. Moreover, the psychometric properties are context-specific and therefore the results are limited to the patients diagnosed with shoulder tendonitis only.

The test-retest reliability of the DASH-H assessed using the ICC values was 0.86. There is a substantial body of evidence attesting the test-retest reliability of the English DASH and its translated versions. In general, the reliability assessed by weighted ICC is known to lie somewhere between 0.77 and 0.98 in patients with shoulder pathology [5]. The ICC values specifically for the shoulder tendonitis patient group have been shown to be 0.91 [24]. The ICC value is the ratio of relationship between within subjects and between subjects variances over the test and retest intervals. Therefore, the change in the patient's status between these intervals can affect the ICC and in effect the level of reproducibility of the test scores. The participants showed significant improvement between occasion 2 and occasion 1 (mean change of 10.4 ± 6.1; *p* = 0.004). This may have adversely influenced the reliability estimates of the DASH-H, which despite being excellent (ICC = 0.86) may have been better had the patients remained clinically stable between the test-retest intervals. There is no defined test-retest interval for assessing the reliability. However, it is suggested that the test-retest interval has to be shorter for individuals with acute musculoskeletal problems since their clinical status will change rapidly yielding imprecise reliability estimates. The test-retest interval in our study was 2-3 days which is short and should not have affected the reliability estimates especially in patients with atraumatic shoulder tendonitis which is not considered an acute condition. There is a strong possibility that the smaller sample recruited in this study would likely have affected the between subjects variance. Since the reliability estimate of ICC is derived from the ratio of within subjects and between subjects variances, unstable between subjects variance can adversely affect the reliability estimates.

The DASH-H showed moderate relationships with the VAS scales, where the relationship with the VAS-D was slightly better compared to that with VAS-P. This moderate relationship was expected since the VAS scales examined the pain/disability over a single question with a reference period of 24 hours, whereas the DASH-H examined the disablement resulting from shoulder tendonitis using 30 UE functional tasks with a 7-day reference period. The strength of the convergent relationships between the measures is based on the similarities in the scope and breadth of the construct being assessed by these measures. The VAS-P examines shoulder pain, while the DASH-H, though having a question on arm pain, is not directly intended to assess pain but characterize disability in upper extremity functions. Therefore, the lack of convergence with the VAS-P scale and the moderate relationship (*r* = 0.41) is expected.

The excellent responsiveness estimates of the DASH-H (ES and SRM of 2.2 and 6.1, resp.) are strongly indicative of the utility of the DASH-H as an important tool to examine change in patient's status with treatment. The SRM and ES reported in this study are higher compared to the previous study that reported ES/SRM values of 1.06/1.08 in patients with shoulder tendonitis [24]. The high ES is a function of mean change between the test occasions divided by the variability in the DASH scores. Similarly, the SRM is the function of mean change between the test occasions divided by the variability in the change score between the two occasions. The cohort in this study had significant change in functional status between occasion 1 and occasion 3 leading to a large shift in the DASH-H scores (mean change of 31.9 ± 5.2; *p* < 0.001). The variability of the DASH scores at baseline was higher (SD = 14.3); however, the variability in the change scores between occasion 1 and occasion 3 was very small (SD = 5.2) which led to higher SRM. In general, the ES/SRM values are dynamic and vary based on characteristics of patients. Nonetheless, based on the results of this study and those of the previous study [24], it is evident that the DASH is highly responsive to change in patients with shoulder tendonitis.

MDC_90_ and MDC_95_ for the DASH-H found in this study were 12.4 and 14.7, respectively. MDC_90_ represents the change in the DASH score required over the follow-up assessment which suggests a true change in the patient's status at 90% confidence interval. For example, if a patient scored 60/100 on the DASH at the initial assessment session, any score of ≤47.6/100 (considering 12.4 as MDC_90_ of the DASH) would suggest that there is a real change in patient's status. Similar to other data discussed above, we could find only one study that has examined these MDC values in patients with shoulder tendonitis [24]. Interestingly, Schmitt and Di Fabio [24] found MDC_90_ value for the English DASH to be 12.2 which is very similar to the one found in this study. For all shoulder pathologies, the weighted MDC_90_ and MDC_95_ for the DASH are 10.5 and 10.8, respectively [5]. MDC values are derived from the SEM, which in turn is dependent upon the variability in the DASH scores at baseline and ICC values. As discussed earlier, the ICC values in our study could have been better had the patients remained clinically stable between the test-retest intervals. One possibility is that this change in clinical status between intervals affected MDC values which ended up being higher than the weighted averages. Another scenario is that since the MDC values were similar to the previous study in the same population, they are more realistic for this patient population. Lastly, we emphasize that the MDC values are the function of variability in the population between test occasions and may not reflect clinically meaningful change to the patient. The minimal clinically important difference is a more appropriate indicator of meaningful change which we did not calculate in this study.

The main limitation of this study is the smaller sample size recruited in analyzing the psychometric properties of the DASH-H. Firstly, we acknowledge that we did not perform sample size calculations to determine how many patients we would have needed to sufficiently power all the analyses conducted in this study. The reason for that was the preliminary nature of analyses and the fact that we were trying to pilot-test the properties of a newly translated tool versus assessing the properties of an established measure. In view of this, we acknowledge that the generalizability of the estimates of psychometric properties given in this study should be limited. Secondly, the scope of psychometric properties was limited in a sense that we have not established the factor structure of DASH-H. The DASH is believed to assess a single factor of functional ability. However, several research studies have questioned this hypothesized unidimensional structure of the English DASH [25–27]. The unidimensional structure of the DASH-H can be examined in future trials. Lastly, there are many dialects of Hindi language and the translated version described in this study and available on the DASH website [11] may not represent all the possible dialects of Hindi spoken across India and other parts of the world. In view of this, it is imperative that future research focuses on validating this version of DASH-H across different geographic and linguistic regions of India and parts of the world where Hindi speaking populations reside.

In conclusion, this study translated and tested the psychometric properties of the Hindi version of the DASH which is one of the most commonly used UE musculoskeletal outcome measures of functional disability in rehabilitation practice. While the results are limited by smaller and exclusive groups of patients with shoulder tendonitis, the robust methods of data analyses and estimates of psychometric properties will trigger future research in validating the DASH-H in different clinical subgroups involving upper extremity. The results of this study do support the DASH-H as a reliable, valid, and responsive measure in assessing functional disability in Hindi speaking patients with shoulder tendonitis.

## Figures and Tables

**Table 1:** Characteristics of the participants (*N* = 30).

Parameter	Statistic
Age (years) (mean ± SD)	53.3 ± 6.9
Side of injury (right/left)	15/15
Hand dominance (right/left)	26/4
DASH-H score	
Occasion 1	46.3 ± 14.3
Occasion 2	35.9 ± 10.7
Occasion 3	18.1 ± 7.1
VAS-pain	
Occasion 1	6.8 ± 1.2
Occasion 2	4.9 ± 0.9
Occasion 3	2.2 ± 1.2
VAS-disability	
Occasion 1	6.1 ± 1.5
Occasion 2	4.2 ± 0.9
Occasion 3	2.3 ± 0.9
AROM-shoulder flexion	
Occasion 1	98.9 ± 21.9
Occasion 2	117.3 ± 17.5
Occasion 3	147.1 ± 16.1
AROM-shoulder abduction	
Occasion 1	85.6 ± 20.8
Occasion 2	106.3 ± 16.8
Occasion 3	135.5 ± 15.8
AROM-shoulder external rotation	
Occasion 1	22.8 ± 11.1
Occasion 2	31.1 ± 9.7
Occasion 3	43.8 ± 12.5

SD, standard deviation; DASH-H, Hindi version of the Disabilities of Arm, Shoulder, and Hand questionnaire; VAS, visual analogue scale.

**Table 2:** Reliability and internal consistency (*N* = 30).

	ICC (95% CI)	CA
DASH-H	0.86 (0.68, 0.95)	0.87
VAS-P	0.75 (0.47, 0.88)	—
VAS-D	0.37 (0.06, 0.71)	—
AROM-flexion	0.72 (0.42, 0.87)	
AROM-abduction	0.60 (0.15, 0.81)	
AROM-ext. rotation	0.77 (0.52, 0.89)	

DASH-H, Hindi version of the Disabilities of Arm, Shoulder, and Arm questionnaire; ICC, Intraclass correlation coefficient; CI, confidence interval; CA, Cronbach's alpha; VAS-P, visual analogue scale-pain; VAS-D, visual analogue scale-disability.

**Table 3:** Correlation between the measures (*N* = 30).

	VAS-pain	VAS-disability	AROM-Flex	AROM-Abd	AROM-ER
DASH-H total	0.41^*∗*^	0.51^*∗∗*^	−0.71^*∗∗*^	−0.31	−0.47^*∗∗*^
VAS-pain		0.45^*∗∗*^	−0.27	−0.37^*∗*^	−0.2
VAS-disability			−0.14	−0.09	−0.41^*∗*^
AROM-Flex				0.66^*∗∗*^	0.48^*∗∗*^
AROM-Abd					0.02

DASH-H, Disabilities of Arm, Shoulder and Hand-Hindi; AROM, active range of motion; Flex, flexion; Abd, abduction; ER, external rotation.

^*∗*^Correlation significant at *p* < 0.05.

^*∗∗*^Correlation significant at *p* < 0.01.

*r* values in bold indicate moderate or high correlation.

**Table 4:** Responsiveness and other statistics between occasion 1 and occasion 3 (*N* = 23).

	DASH-H	VAS-P	VAS-D
Effect size	2.2	3.8	2.64
SRM	6.1	3	2.73
SEM	5.3	0.6	1.2
MDC_90_	12.4	1.5	2.8
MDC_95_	14.7	1.7	3.3

DASH-H, Hindi version of the Disabilities of Arm, Shoulder, and Hand questionnaire; SRM, standardized response means; SEM, standard error of measurement.
